# Commentary: Cognitive Behavioral Therapy vs. Eye Movement Desensitization and Reprocessing for Treating Panic Disorder: A Randomized Controlled Trial

**DOI:** 10.3389/fpsyg.2018.01061

**Published:** 2018-06-25

**Authors:** Giampaolo Perna, Erika Sangiorgio, Massimiliano Grassi, Daniela Caldirola

**Affiliations:** ^1^Department of Clinical Neurosciences, Hermanas Hospitalarias, Villa San Benedetto Menni Hospital, FoRiPsi, Como, Italy; ^2^Italian Association for Behavior Analysis and Modification and Behavioral and Cognitive Therapy, Milan, Italy; ^3^Department of Psychiatry and Neuropsychology, Faculty of Health, Medicine and Life Sciences, Maastricht University, Maastricht, Netherlands; ^4^Department of Psychiatry and Behavioral Sciences, Leonard Miller School of Medicine, Miami University, Miami, FL, United States

**Keywords:** EMDR, Psychotherapy, non-inferiority, cbt, panic disorder

We read with interest the article written by Horst et al. (2017) exploring whether or not Eye Movement Desensitization and Reprocessing (EMDR) can be considered to be as effective a treatment method as Cognitive-Behavior Therapy (CBT) for patients with Panic Disorder (PD). Using the Agoraphobic Cognition Questionnaire (ACQ), the Body Sensations Questionnaire (BSQ), and the World Health Organization Quality of Life short version (WHOQOL-Bref) as measures, the authors did not find EMDR inferior to CBT in the following actions: reducing anxiety related cognitions, alleviating the fear of bodily sensations, and improving most aspects of the quality of life. The authors considered their results inconclusive with regard to agoraphobic avoidance as measured through the use of the Mobility Inventory (MI). Based on these results, the authors confirmed “EMDR therapy proved to be as effective as CBT for treating PD patients.” Although the study is interesting, we think that the above-quoted conclusion is inappropriate and overstated because of two main methodological reasons.

First, we think that the authors did not use the appropriate outcome measures to compare therapies for treating PD. PD refers to recurrent unexpected panic attacks (PAs) (American Psychiatric Association, 2013), which represent the hallmark of the disorder. The assessment of presence, frequency, and severity of PAs is essential both for its accurate diagnosis and evaluation of the effectiveness of treatments over time (American Psychiatric Association, 2013; Perna and Caldirola, 2017). In line with this belief, evidence-based guidelines (Furukawa et al., 2009) have proposed several appropriate instruments, such as the Panic-Associated Symptoms Scale (Argyle et al., 1991) or the Panic Disorder Severity Scale (Shear et al., 1997) with response/remission criteria based on clinical changes in panic severity that necessarily include the evaluation of PAs. Regrettably, the study under review is completely lacking in the assessment of PAs. The authors included only measures assessing the cognitive misinterpretation of anxiety (ACQ), fear related to bodily sensations (BSQ), and severity of agoraphobia (MI). While the first two aspects deserve evaluation as they can be present in patients with PD, they should not be considered as primary outcome measures of the disorder and they cannot replace the assessment of PAs. Thus, the finding of non-inferiority of EMDR to CBT in reducing ACQ and BSQ scores does not prove that EMDR is as effective as CBT in treating PD. The study only demonstrates that these two interventions may have similar efficacy with regard to some cognitive aspects of the disorder. Similarly, although agoraphobia is often an additional diagnosis to PD and can worsen the global severity of the disorder, approximately 20–30% of patients with PD do not have comorbid agoraphobia (as the authors also found in their sample). Further, the two disorders are considered as distinct conditions with non-overlapping diagnostic criteria (American Psychiatric Association, 2013). Thus, the severity of agoraphobia, as measured through MI scores, should not be used as a direct “measure of severity of PD.” It should be seen a measure of a condition that can be related to PD, whose assessment should be associated to that of the PD itself. Having said this, the adjusted analysis concerning MI scores did not conclusively find that EMDR was not inferior to CBT. Thus, this study does not support the use of EMDR for treating comorbid agoraphobia in patients with PD. To summarize, the design of the study was inappropriate for the purpose of drawing reliable conclusions about the treatment of PD. It may only offer suggestions about some aspects associated with the disorder.

Second, the authors did not exhaustively describe the method they have employed in determining the non-inferiority (NI) margins of outcome measures. NI margins should be carefully selected in NI trials and they should be thoroughly justified in the study protocol (Piaggio et al., 2012). Indeed, this is probably the most crucial aspect of designing a NI study as the validity and interpretation of the results rely significantly on the choice of the NI margins. If the latter are questioned, then the entire NI study is questioned as well. As affirmed by several guidelines, such as the FDA and EMEA (European Medicines Agency, 2006; U.S. Department of Health and Human Services Food and Drug Administration, 2016), the selection of NI margins should be based on a combination of statistical reasoning and clinical judgment through a comparison of prior studies conducted on the effect of the intervention under examination. On the basis of these suggestions, the NI margins of this study (ACQ and BSQ, δ = 5; MI, δ = 8; WHOQOL-Bref, δ = −1) are questionable. With regard to the ACQ, BSQ, and MI questionnaires, the authors only reported that the NI margins “were determined by clinical experts” without further detailing their choice and/or justifying their approach by citing an adequate reference. The choice for WHOQOL-Bref was also not totally appropriate since the authors decided on their NI margins on the basis of a prior study by Den Oudsten et al. (2013). That research initiative was conducted in a non-psychiatric population of women affected by breast cancer or by benign breast problems.

In conclusion, given these crucial methodological issues, the statement of the authors that “EMDR proved to be as effective as CBT for treating PD” is not demonstrated by their results, and this study appears unsuitable as a support for the use of EMDR in PD.

## Author contributions

ES and MG drafted the manuscript; GP and DC revised the manuscript for important intellectual content; GP, ES, MG, and CD approved the final version of the manuscript.

### Conflict of interest statement

The authors declare that the research was conducted in the absence of any commercial or financial relationships that could be construed as a potential conflict of interest.
